# Going Deep: Cautious Steps toward Seabed Mining

**DOI:** 10.1289/ehp.123-A234

**Published:** 2015-09-01

**Authors:** Charles W. Schmidt

**Affiliations:** Charles W. Schmidt, MS, an award-winning science writer from Portland, ME, has written for *Discover Magazine*, *Science*, and *Nature Medicine*.

The deep ocean was once assumed to be lifeless and barren. Today we know that even the deepest waters teem with living creatures, some of them thought to be little changed from when life itself first appeared on the planet. The deep ocean is also essential to the earth’s biosphere—it regulates global temperatures, stores carbon, provides habitat for countless species, and cycles nutrients for marine food webs.^1^

Currently stressed by pollution, industrial fishing, and oil and gas development, these cold, dark waters now face another challenge: mining. With land-based mineral sources in decline, seabeds offer a new and largely untapped frontier for mineral extraction, and companies are gearing up to mine a treasure trove of copper, zinc, gold, manganese, and other minerals from the ocean floor.^2^^,^^3^

**Figure:**
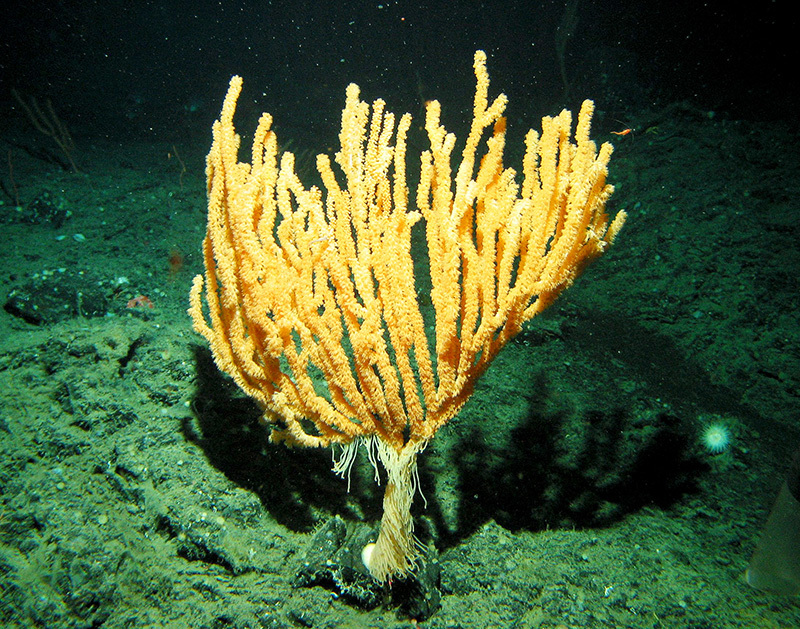
The ocean floor is home to a wealth of species, some of which—such as this bamboo coral (Isidella tentaculum)—have only recently been discovered. Proponents of seabed mining claim it causes less ecological damage than terrestrial extraction. But some researchers are concerned that seabed mining could overwhelm deep-sea ecosystems, adding to concerns about the health of the oceans. © NOAA

Scientists, regulators, and mining companies are now collaborating on frameworks and strategies for mining the seabed responsibly. Cindy Van Dover, director of the Duke University Marine Laboratory and chair of the school’s Division of Marine Science and Conservation, says that’s encouraging, given that seabed mining appears to be inevitable.

“There’s been a lot of engagement on the environmental side,” Van Dover says. “A hundred years from now, people will look back and ask if we got this right. We need to be sure that we do.”

Copper grades, or the percentage of copper per unit of mined substrate, have declined with steadily rising extraction, from a high of 10–20% during the late nineteenth century to less than 1% today.^4^ By contrast, copper grades in seabeds slated for exploitation in 2018 by the Canadian mining company Nautilus Minerals, lying under 1,600 m of water off Papua New Guinea, average 7.2%.^5^ It’s estimated that 500 billion metric tons of polymetallic nodules—mineral clumps loaded with varying levels of manganese, cobalt, nickel, and copper—lie scattered under waters up to 6,000 m deep in the Pacific, Atlantic, and Indian oceans.^6^

Proponents of seabed mining assert that extracting minerals from the deep ocean will inflict less environmental damage than mining on land, which displaces communities, removes entire ecosystems, exacerbates erosion, and pollutes groundwater, rivers, and streams. But according to Craig Smith, a professor of biological oceanography at the University of Hawai‘i at Mānoa, seabed mining will also stir up vast plumes of sediments, some of which could resettle over areas much larger than the mine sites themselves. Scientists worry the plumes could cause widespread ecological damage and kill off deep-sea fauna that they know little about. Without appropriate regulations, they say, seabed mining will further erode the ocean’s capacity to provide essential ecological services, adding to what are already acute concerns for the ocean’s overall health.

“Deep-ocean ecosystems can be incredibly fragile,” Smith says. “And it’s possible that after the mining starts, huge areas could be impacted before any one of them has a chance to bounce back.”

## ISA in the Decision Seat

To a large extent, environmental prospects for seabed mining hinge on the deliberations of a group called the International Seabed Authority (ISA). The ISA was created by the United Nations Convention on the Law of the Sea (UNCLOS), a treaty ratified by most of the world’s nations (although not by the United States). The UNCLOS governs the use and protection of seabed resources.^7^ Within that context, the ISA has a mandate to organize, regulate, and control all mineral-related activities in what’s known as “the Area,” or the international seabed lying beyond the exclusive economic zones (EEZs) of specific countries. Any coastal nation may claim an EEZ up to 200 nautical miles (370 km) off the country’s shore, within which the country is responsible for regulating mining.^8^

The UNCLOS defines the Area as a “common heritage of mankind” that is not subject to direct claims by sovereign states. The ISA administers this heritage by issuing mining leases in the Area to countries or corporations that will, in turn, be obligated to pay mining royalties back to the ISA. Because the royalties will come from mining a “common heritage,” the ISA will then redistribute the money to countries in the developing world using procedural mechanisms that are still being developed.

**Figure:**
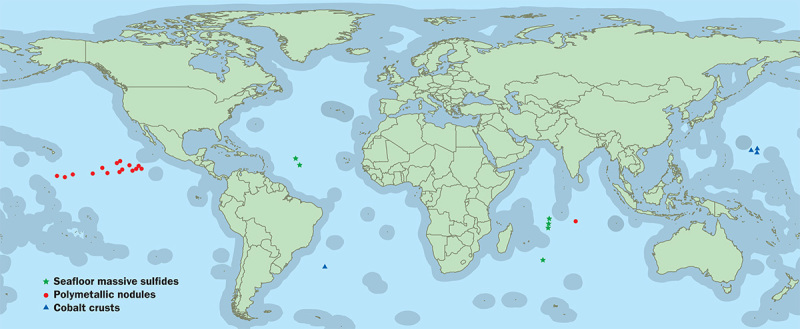
So far, the International Seabed Authority has issued 26 exploratory leases in sections of “the Area”—the vast international seabed that lies outside individual countries’ borders (indicated on the map in gray). The exploratory leases issued to date cover approximately 2 million km^2^ of seabed. No exploitation leases have been issued yet. Jane Whitney/janewhitney.com

Given the inherent tension between the ISA’s dual mandates to collect and distribute royalties from mining licenses and to protect the marine environment, skeptics have described the organization as a “fox in the henhouse.” Michael Lodge, the ISA’s legal counsel and deputy to its secretary-general, responds that “the system is full of checks and balances, with different interest groups in different chambers.”

Before the UNCLOS came into force in 1994, a so-called pioneer regime was established under the United Nations with the authority to issue “pioneer claims” to enterprises that had already invested in minerals exploration. Lodge says six pioneer claims for minerals exploration were issued in 1984, each totaling an area of 75,000 km^2^. Those claims transferred into official leases when the ISA became a legal entity 10 years later.

Between 1984 and 2011, Lodge says, the ISA issued no further leases, but then the numbers started surging, coincident with completion by the ISA of regulations for exploration.^9^ According to Lodge, the ISA has so far issued 26 exploratory leases covering a total of approximately 2 million km^2^ of seabed. Exploitation leases to actually extract minerals will follow when the corresponding regulations are final.

According to Maurice Tivey, a geologist and senior scientist at the Woods Hole Oceanographic Institution, two converging factors are driving the spike in exploration. One of them is technological innovation leveraged from the oil and gas industries, which are migrating steadily toward the deep ocean. The other factor is a projected surge in demand especially for copper, but also for other minerals, including “rare earth” minerals used in hybrid car components, smart phones, computers, solar panels, and many other electronic devices. Duncan Currie, a legal and political advisor with the Deep Sea Conservation Coalition, headquartered in Amsterdam, the Netherlands, says countries and corporations are taking a long view on seabed mining, anticipating mineral shortages and higher prices that will eventually make the practice cost-effective.

## Types of Deep-Sea Minerals

Desirable minerals are found in three types of seabed deposits. Located in comparatively shallower waters 1,500–2,500 m deep, the most accessible deposits are called seafloor massive sulfides (SMS). They occur where seawater percolates down through fissures in the earth’s crust—at volcanically active zones called midocean ridges (where tectonic plates diverge) and at submarine volcanic chains.^2^ Cold seawater reacts with hot rock beneath these geologic features, resulting in hydrothermal vents that spew super-heated fluids into the water column. In some cases, hydrothermal vents appear as “black smokers,” chimney-like structures discharging dark clouds of sulfur-bearing material that accumulates into SMS deposits. These deposits typically contain high levels of copper and zinc, as well as gold and silver.^10^

**Figure:**
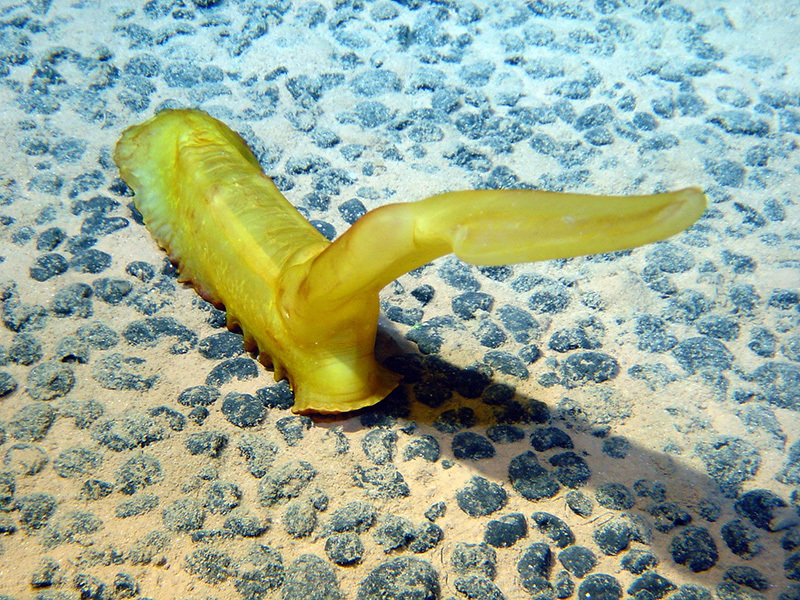
Polymetallic nodules—seen here with Psychropotes longicauda, a species of sea cucumber—dot the abyssal plains that cover nearly two-thirds of the earth’s surface. These are some of the several billion metric tons of recoverable nodules estimated to lie in the Clarion–Clipperton Fracture Zone. © Lenaick LEP (image license available at http://creativecommons.org/licenses/by-nc/4.0/legalcode)

**Figure:**
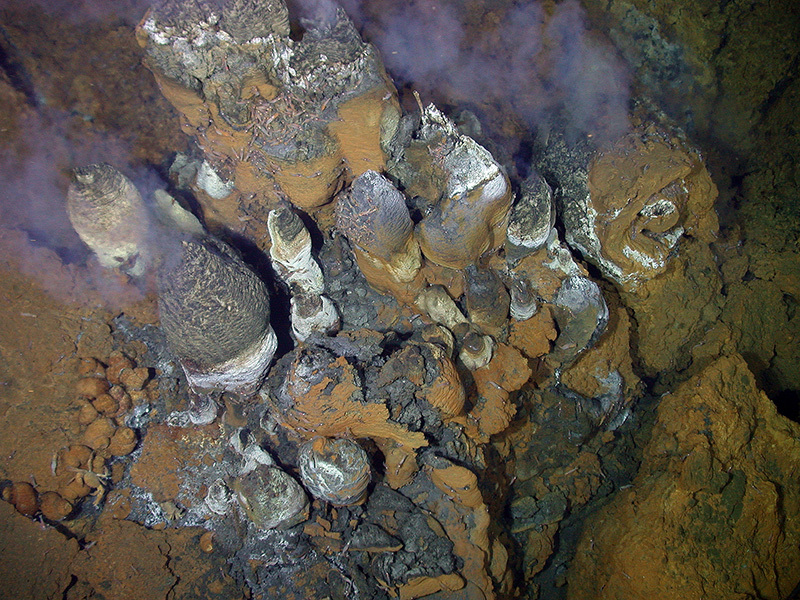
SMS deposits form around hydrothermal vents known as black smokers. The dark “smoke” is actually fluid expelled from the vents; minerals in this fluid settle around the base of the vent mound. This black smoker is located in the Eastern Manus Basin off Papua New Guinea. Photo courtesy of Maurice Tivey and the WHOI Deep Submergence Laboratory, Cruise Manus 2006 with ROV Jason-2

**Figure:**
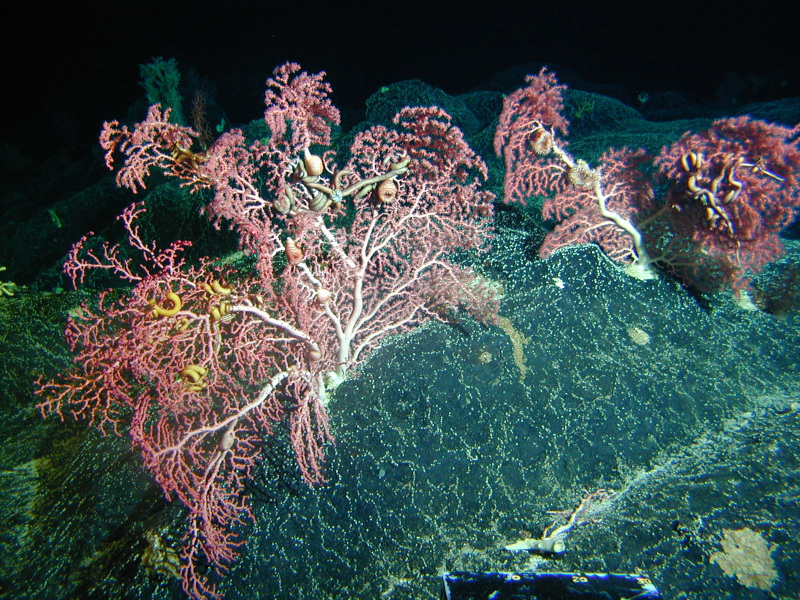
Cobalt crusts, seen here with colonies of bubblegum coral (Paragorgia arborea) at a depth of 350 m, are found on undersea mountains swept by high currents. Japan is the only country that has invested substantially in technologies to extract these deposits. © NOAA

Polymetallic nodules are much more widespread deposits. They are spread across the abyssal plains, which cover an estimated 60% of the earth’s surface.^3^ These vast, flat expanses of the ocean floor lie an average of 3,000–4,000 m underwater.^3^ Eighty percent of the exploratory leases for these nodules are located in a vast region called the Clarion–Clipperton Fracture Zone (CCZ), which extends from Mexico to Hawaii and ranges from 4,000 to 5,000 m in depth. The CCZ is estimated to contain several billion metric tons of recoverable nodules, each roughly 5–10 cm in size, lying half-buried on the seafloor.^6^

Cobalt-rich crusts make up the third class of seabed mineral deposits. These crusts are found on undersea mountains, or “seamounts,” in shallower waters; most of the mineable crusts are at a depth of 700–2,500 m. Cobalt crusts are formed in areas where iron and manganese has precipitated from seawater over millions of years. They’re also loaded with cobalt, nickel, tellurium, and rare earth metals that aggregate in concentrated layers up to 25 cm thick on hard rock surfaces.^11^

**Figure:**
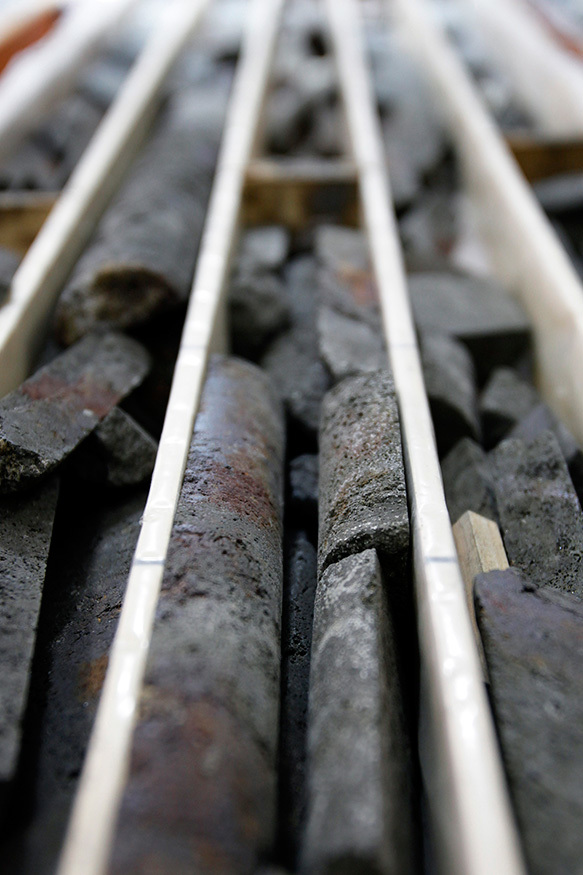
SMS core samples await geologic analysis in a Japanese laboratory. Deep-sea deposits can be rich sources of precious metals, rare earths, and other valuable minerals. © Kiyoshi Ota/Bloomberg via Getty Images

The different mineral types are surrounded by a variety of fauna. Some SMS sites have low biodiversity, but others are populated by a rich assemblage of species, including tubeworms, clams, snails, shrimp, crabs, and cold-water corals. The bacteria and other single-celled organisms at the bottom of hydrothermal vent food chains are chemosynthetic, meaning they derive energy from oxidation of inorganic molecules instead of from sunlight, as occurs with photosynthesis.^12^

“It’s possible that all life on earth emerged from these hydrothermal systems,” says Richard Steiner, a marine conservation biologist and consultant based in Anchorage, Alaska. “And since there are only [an estimated] five hundred to five thousand hydrothermal vent systems in the world ocean,^13^ each one averaging a square kilometer each, they’re also extremely rare.”

Scientists point out that SMS ecosystems evolved to recover quickly from violent disturbances. Indeed, the Solwara 1 site lies within 500 m of an active volcano that, according to unpublished findings from Tivey and colleagues, deposited 6 million tons of fresh sediments between 2005 and 2011. However, mining has also been proposed for inactive vent sites, which may have lost some of this resiliency and thus may be likely to recover much more slowly, says Lisa Levin, a professor at the Scripps Institution of Oceanography.

Scientists know little about the benthic (deep-sea) species residing in the abyssal plains, but what they’re learning shows them to be highly adapted to an extreme environment, where temperatures hover just above freezing and pressures become crushing.^14^ Studies show much of the fauna to be limited in size, slow to mature and with low rates of metabolism, reproduction, and colonization.^3^

Moreover, the addition of new sediments in abyssal plains depends on the gradual rain of particles from the sea surface. These include the remains of dead plankton and other organisms, plus tiny amounts of wind-blown grains of inorganic minerals, mainly quartz. New sediments accumulate in abyssal plains at an average rate of just 2–3 cm per thousand years, according to Philip Weaver, managing director of Seascape Consultants, Ltd., in Romsey, United Kingdom. And in the deepest plains, he says, it’s even lower, perhaps 0.5–1 cm over the same time scale.^15^

According to Smith of the University of Hawai‘i, the sluggish biology and low rates of sedimentation virtually ensure that abyssal plain ecosystems won’t recover from mining for hundreds of years. Evidence supporting that view is already available: In 1978, scientists performing an experiment scooped polymetallic nodules from the CCZ and left a track in the sediments that was 1.5 km wide and 4.5 cm deep. When a different research team returned to the same site 26 years later, the track was still clearly visible, analogous to the footsteps left by astronauts on the moon. What’s more, nematode populations in the track were still disturbed, with the abundance and diversity significantly lower than in adjacent areas where nodules had not been removed.^16^

By contrast, the seamounts where cobalt crusts are found tend to be high in biological productivity, Levin says. “The physics is such that you have a lot of water motion, and that favors the growth of corals and fish,” she explains. But these ecosystems also grow slowly, she says; some fish can be more than 100 years old. “At this stage,” Levin says, “we expect these ecosystems will also recover slowly from disturbances.”

## Exploitation

Cobalt crusts, being stuck to rock, could be challenging to remove. Miners will have to somehow recover the crusts without collecting too much rocky substrate, which would dilute the quality of the ore. According to the ISA, only the Japanese have invested substantially in technologies to recover cobalt crusts. Elsewhere, the technology remains in its infancy.^6^

Substrate challenges are less daunting at SMS sites, where remotely operated vehicles will grind and cut their way through mineral deposits up to 30 m thick. These sites also have a relatively small footprint. Nautilus Minerals’ site off Papua New Guinea, for instance, called Solwara 1, reaches 20–25 m into the seabed, yet the site occupies only 0.11–0.14 km^2^ of ocean floor, says Renee Grogan, the company’s environment manager. Grogan says that compared with terrestrial mining, “this is a very small footprint for what we anticipate will be a very large yield of ore.”

Polymetallic nodules, meanwhile, will be “vacuumed” from the top 5–10 cm of sediment on the seafloor.^16^ According to the ISA, polymetallic nodules are only profitable when the yield exceeds 10 kg/m^2^.^6^ By one estimate, a profitable site will mine 1 km^2^ of the seafloor every day, and a mature industry will disrupt up to 12,000 km^2^ around the world every year.^6^ “But then again,” Smith says, “abyssal plains are probably the most widely distributed ecosystems on the planet. So the percentage impacted may be quite small, especially if [extraction is] well managed.”

Regardless of where it occurs, seabed mining will stir up some amount of sediment, creating plumes that in some cases could fall out over areas larger than the mine sites themselves. These plumes could have a variety of potential impacts. Plumes released near the surface may reduce light penetration and temperature and thus impair plankton growth, with rippling effects on the food web. Sediment also might smother benthic organisms as it settles, particularly those living in abyssal plains, which never evolved to cope with such large amounts of sediment sinking from above.^17^

Furthermore, the plumes could be toxic, especially those generated from mining SMS sites, which, according to Duke’s Van Dover, may liberate harmful levels of lead, arsenic, copper, and other elements that were once trapped in the deposits. Van Dover points out that copper is an antifouling agent—“if it’s mobilized in the water,” she says, “then organisms will have to fight off the effects of the contamination.”

**Figure:**
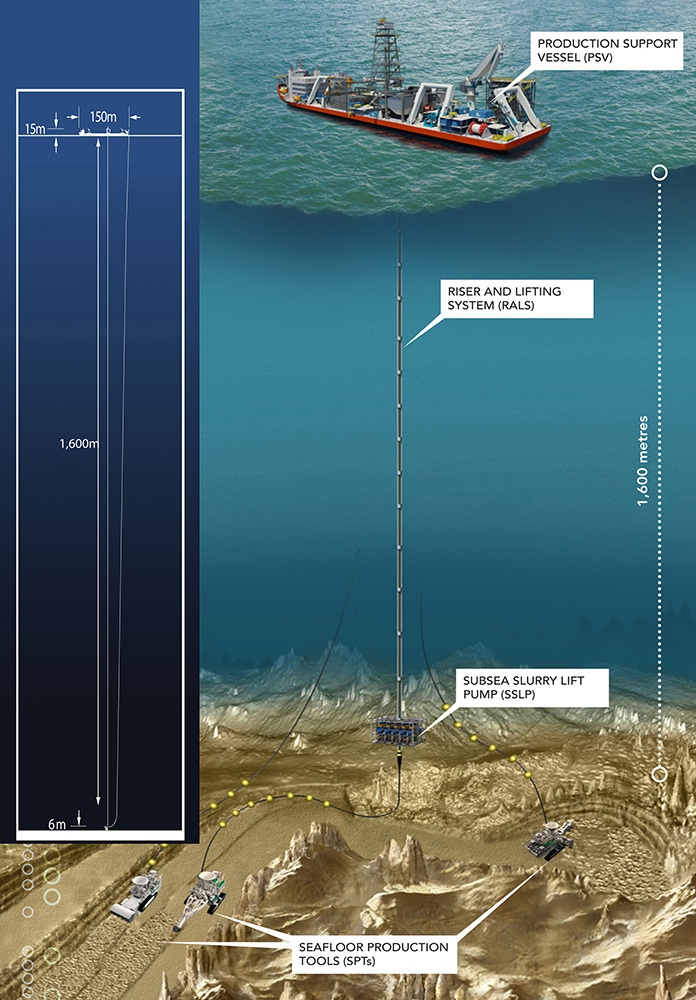
Nautilus Minerals plans to mine SMS deposits using various cutting and collection tools on the seafloor. A slurry of minerals and seawater will be pumped to the surface via a riser and lifting system. The slurry will be dewatered aboard a support vessel and the recovered minerals shipped to shore. In an effort to limit potential ecological impacts, the filtered water will be returned to the seafloor through the riser pipes, providing hydraulic power to the pump as it goes. © Nautilus Minerals

Plumes in some locations could have lesser impacts. According to Grogan, modeling suggests that plumes generated from mining Solwara 1 will deposit within 600 m of the extraction zone, making it “a very small off-site impact.” She adds that Solwara 1 is located next to an active volcano, which produces a significant plume of its own, reducing the impact of mining on organisms that have already adapted to these eruptions.

One research program that’s now studying the possible ecotoxicological effects of seabed mining plumes is MIDAS (Managing Impacts of Deep-Sea Resource Exploitation).^18^ Funded by a three-year grant from the European Commission, MIDAS conducts broad-based research in a number of areas with the aim of developing best practices for the deep-sea mining industry.

According to Nélia Mestre, a postdoctoral research assistant at the University of Algarve, Portugal, who works with the program, much about how the plumes could affect life in the deep ocean remains unknown. High pressure and low temperatures might influence the bioavailability of toxic elements, she says, and deep-sea species may be either less or more susceptible to plume toxicity than species in shallower waters.

**Figure:**
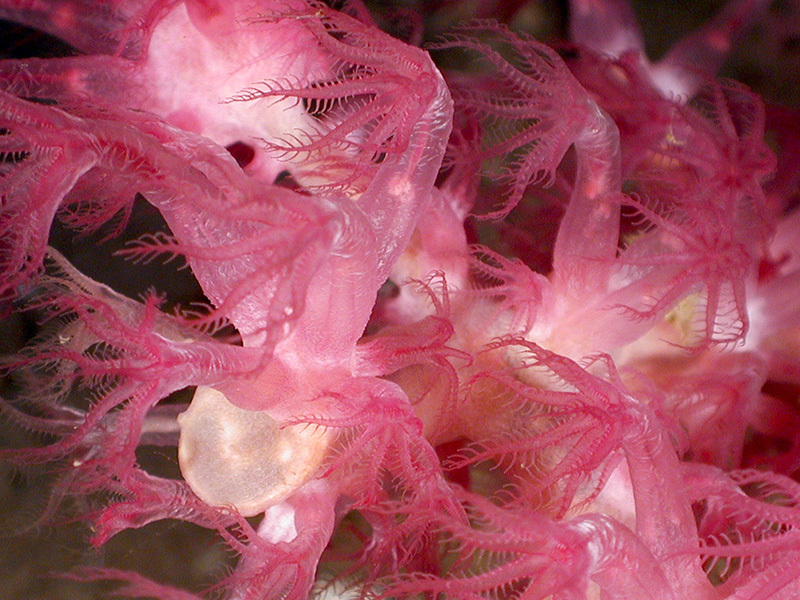
These octocorals live at a depth of 1,500 m in the Gulf of Mexico. The ocean is an essential regulator of the earth’s biosphere, and its deepest waters likely hold direct human health benefits in the form of yet-undiscovered therapeutic substances. Of the researchers studying these waters, Cindy Van Dover says, “We are a new breed of scientists who think about the environmental management of a place that covers most of the planet, a place most people don’t think about from one day to the next.” And while the seafloor still holds many mysteries, Van Dover adds, “What we do know is that the health of the planet depends upon the health of the ocean.” © NOAA

“The tolerance difference could go both ways,” Mestre explains. “For instance, SMS species are adapted to chemicals released by black smokers at levels that could be toxic to shallow-water species. We hope that by the end of the MIDAS project we will have an indication of the potential hazard of chemicals present in plumes to local fauna in comparison to shallow-water fauna.”

## A Framework for Protection

During its July 2015 session, which ran for two weeks in Kingston, Jamaica, the ISA began to consider a draft framework for the exploitation of seabed resources. Also in July, Smith and 10 colleagues published a paper in *Science* recommending a precautionary approach to seabed mining that would emphasize the creation of Marine Protected Areas (MPAs), and calling on the ISA to “[suspend] further approval of exploration contracts (and not approve exploitation contracts) until MPA networks are designed and implemented for each target region.”^8^ Smith argues that MPA networks are needed to guarantee that a significant proportion of the global deep-sea ecosystem remains intact and viable.

A provisional environmental management plan protecting roughly 1.4 million km^2^ was established for the CCZ by the ISA in 2012.^8^ However, an environmental management plan has not been established for regions of the Pacific, Indian, and Atlantic oceans, where the ISA continues to issue exploration leases.

Smith and his coauthors are concerned that MPAs might be spaced too far apart, without the connectivity needed to prevent localized extinctions. “We don’t want to be overly critical of the ISA, but they really need to get these regional MPA plans in place soon,” he says. “Exploration claims in the CCZ are already compromising our ability to create MPAs in some areas.”

In response, Lodge counters, “There is no basis for either suspending contracts or placing a moratorium on exploration, since exploration provides the only means for gathering environmental data. A suspension of exploration would be self-defeating.”

According to Lodge, the ISA is now reaching “saturation on exploration leases.” He says there are perhaps 10 other promising areas that haven’t been leased for exploration yet, but the industry appears to be consolidating around a limited number of projects. If exploitation ultimately succeeds in these areas, he says, then deep-sea mining is likely to experience a huge amount of growth.

The hope among scientists and other environmental stakeholders is that this growth is matched by successful efforts to protect key habitats. Van Dover says these efforts might focus especially on protecting thermal vent communities, which she describes as “beautiful, rare, and important.”

Smith views potential extinctions in moral terms, pointing out that “the deep sea is raw material for evolution—large-scale extinctions would profoundly affect what makes our planet unique.” And like other endangered habitats, such as tropical rainforests, the deep ocean likely harbors untapped biological resources that might one day be used to develop new drugs and other products that benefit humankind.^19^ “We’re talking about the largest and least understood biome on earth,” says Steiner. “And right now very little of it is protected.”
